# Non-coding RNA Regulated Cross-Talk Between Mitochondria and Other Cellular Compartments

**DOI:** 10.3389/fcell.2021.688523

**Published:** 2021-08-03

**Authors:** Jinliang Huang, Sipeng Wu, Pengcheng Wang, Geng Wang

**Affiliations:** ^1^School of Life Sciences, Tsinghua University, Beijing, China; ^2^State Key laboratory for Cellular Stress Biology, Innovation Center for Cell Signaling Network, School of Life Sciences, Xiamen University, Fujian, China

**Keywords:** mitochondria, retrograde signaling, nucleus, non-coding RNAs, trafficking, PNPASE

## Abstract

Mitochondria are the main hubs for cellular energy production. Metabolites produced in mitochondria not only feed many important biosynthesis pathways but also function as signaling molecules. Mitochondrial biosynthesis requires collaboration of both nuclear and mitochondrial gene expression systems. In addition, mitochondria have to quickly respond to changes inside and outside the cells and have their own functional states reported to the nucleus and other cellular compartments. The underlying molecular mechanisms of these complex regulations have not been well understood. Recent evidence indicates that in addition to small molecules, non-coding RNAs may contribute to the communication between mitochondria and other cellular compartments and may even serve as signals. In this review, we summarize the current knowledge about mitochondrial non-coding RNAs (including nucleus-encoded non-coding RNAs that are imported into mitochondria and mitochondrion-encoded non-coding RNAs that are exported), their trafficking and their functions in co-regulation of mitochondrial and other cellular processes.

## Introduction

Mitochondria are organelles originated from ancient proteobacteria through endosymbiosis. Over time, the majority of their endosymbiont genome has been transferred to the host cell nucleus (Calvo et al., 2016). Mitochondria, however, maintain a small compact genome (Anderson et al., 1981). Mitochondrial biosynthesis thus requires collaboration of both nuclear and mitochondrial gene expression systems. Maintaining a small mitochondrial genome thereby provides mitochondria and the host cells a new layer of regulation and control by coordinating mitochondrial functional states with nuclear and other intracellular, and even extracellular events.

Through evolution, some other bacterial characteristics of mitochondria have also been preserved. Mitochondria still retain their own transcriptional and translational machineries, and the mitochondrial ribosomes resemble bacterial ribosomes in many ways (Brown et al., 2014; Greber et al., 2015; Rorbach et al., 2016). In addition, the fission of mitochondria and bacterial division share certain similarities, even though mitochondria have two membranes: the outer membrane and the inner membrane (Friedman and Nunnari, 2014). In a sense, some materials within mitochondria are still treated as foreign objects by the host cells. For example, mitochondrial DNA (mtDNA) could trigger the host cell immune responses when leaked into the cytosol, activating the cGAS-STING pathway (Oka et al., 2012).

Mitochondrial genome is circular, and in humans encodes 22 tRNAs, 2 rRNAs, and 13 OXPHOS proteins (Anderson et al., 1981). mtDNA is transcribed into poly-cistronic preRNAs. Processing and modification of the nascent RNAs yields functional mitochondrial mRNAs, rRNAs, tRNAs, and some other non-coding RNAs (Masters et al., 1987). Biosynthesis of mitochondria, nevertheless, depends largely on the nuclear genome, and the majority of mitochondrial proteins are synthesized in the cytosol or on the outer membrane of mitochondria, imported and then assembled into functional proteins and complexes (Schmidt et al., 2010; Richter-Dennerlein et al., 2015). Increasing evidence shows that various types of nucleus-originated RNA species can also translocate to mitochondria from the cytosol (Leucci et al., 2016; Kim et al., 2017; Vendramin et al., 2018; Gusic and Prokisch, 2020; Sang et al., 2021).

Recent studies have also shown that translocation of RNAs across mitochondrial membranes appears to be bidirectional: the imported RNAs or mitochondrion-transcribed RNAs can also be exported to the cytosol to regulate important cellular processes (Eduardo et al., 2011; Cheng et al., 2018; Dhir et al., 2018; Zheng et al., 2019).

Although more and more nuclear gene-encoded RNAs have been found in mitochondria, most of their functions remain unclear, and their translocation molecular mechanisms are still to be further uncovered. The first component of the RNA import machinery, polynucleotide phosphorylase (PNPASE) was identified in 2010 (Wang et al., 2010). Some progress has since been made on understanding the molecular mechanisms of mitochondrial RNA translocation and the functions of some of these mitochondrial non-coding RNAs, which we have summarized in this review.

## Mitochondrial RNA Import

Mitochondrial RNA import has been reported in many species, and the importomes seem to vary between species. Most of the studied species, however, seem to import one or more tRNAs. Trypanosomatids (Crausaz Esseiva et al., 2004) and apicomplexans (Esseiva et al., 2004) of parasitic protozoa completely lack tRNA genes in their mtDNAs. Therefore, all the tRNAs within their mitochondria are imported from the cytosol. The mtDNAs of some land plants and protists lack one or several tRNA genes, so tRNA import into mitochondria from the cytosol is also essential for mitochondrial translation (Akashi et al., 1998). Although yeast and mammalian mitochondria encode a full set of tRNAs, a few cytosolic tRNAs have been found within their mitochondria. In yeast, two cytosolic tRNAs, *tRNA^*Lys*^_*CUU*_* (Tarassov et al., 1995b) and *tRNA*^*Gln*^ (Rinehart et al., 2005), are imported into mitochondria, which may play a role in stress response. In mammals, mitochondrial localization of nucleus-encoded *tRNA^*Gln*^_*CUG*_* and *tRNA^*Gln*^_*UUG*_* has been reported (Rubio et al., 2008).

In addition to tRNAs, some nuclear or cytosolic non-coding RNAs have also been found in mammalian mitochondria, such as *5S* rRNA, *H1* RNA, *RMRP*, *SAMMSON*, *TERC*, *GAS5*, siRNA (Gao et al., 2021), pre-miRNAs (Barrey et al., 2011), and miRNAs (Table 1). Their individual regulations and functions have been expertly summarized in previous reviews (Vendramin et al., 2017; Jeandard et al., 2019). In this review, we focus mainly on their translocation mechanisms and their functions in communication between mitochondria and other cellular compartments.

**TABLE 1:** Mammalian mitochondrial RNA importome.

**RNA**	**Function outside mitochondria**	**Proposed function in mitochondria**	**References**
*5S* rRNA	Component of the cytosolic ribosome; assisting rhodanese import	Related to mitochondrial translation?	Magalhães et al., 1998; Smirnov et al., 2010; Smirnov et al., 2011; Wang et al., 2010; Yoshionari et al., 1994
*H1* RNA	Component of the nuclear RNase P required for pre-tRNA processing	Pre-tRNA processing?	Ellis and Brown, 2009; Kikovska et al., 2007; Puranam and Attardi, 2001; Rossmanith et al., 1995; Turk et al., 2013; Wang et al., 2010
*RMRP*	*5.8S* rRNA processing	Involved in mitochondrial RNA metabolism?	Chang and Clayton, 1987b; Chang and Clayton, 1987a; Lu et al., 2010; Noh et al., 2016; Wang et al., 2010
*SAMMSON*	Facilitating p32 targeting to the mitochondria in melanoma cells; regulating rRNA maturation and protein synthesis	Unknown	Leucci et al., 2016; Vendramin et al., 2018
*TERC*	RNA component of telomerase	Mitochondrion-cytosol communication	Cheng et al., 2018; Zheng et al., 2019
*GAS5*	Regulating *INSR* gene transcription; functioning as an RNA sponge	Modulating mitochondrial tricarboxylic acid flux	Mourtada-Maarabouni et al., 2010; Sang et al., 2021
Various miRNAs (including miR-1, miR-181c, miR-378) pre-miRNAs, and siRNAs	Repressing mRNA translation	Repressing or activating mRNA translation; repressing transcription	Barrey et al., 2011; Gan et al., 2019; Gao et al., 2021; Jeandard et al., 2019; Vendramin et al., 2017; Zhang et al., 2014
*28S* rRNA	Component of the cytosolic ribosome	None (degraded in the mitochondrial IMS)	Huang et al., 2018

Based on the existing data on RNA import into mitochondria, the mechanisms seem to be quite diverse, with different RNAs require different specific factors.

The mitochondrial RNA import process can be roughly divided into three steps:

### The Step(s) Before Cross-Membrane Translocation

Cytosolic factors appear to be required for mitochondrial import of some non-coding RNAs but not all. In *Trypanosoma brucei*, the tRNA T-stem nucleotide pair and its cytosolic binding partner elongation factor 1a (EF1a) determine the specificity of tRNA import into mitochondria (Bouzaidi-Tiali et al., 2007). On the other hand, isolated mitochondria from *Leishmania tarentolae* can import *in vitro*-transcribed tRNAs without any added cytosolic factors (Rubio et al., 2000). Potato (*Solanum tuberosum*) mitochondria can also import cytosolic *tRNA*^*Ala*^ of *Arabidopsis thaliana* in the absence of additional cytosolic protein fraction (Delage et al., 2003).

In *Saccharomyces cerevisiae*, one of the two cytoplasmic lysine-tRNA isoacceptors, *tRNA^*Lys*^_*CUU*_* (*tRK1*), is selectively imported into mitochondria. Mitochondrial outer membrane-attached glycolytic enzyme enolase binds the aminoacylated *tRNA^*Lys*^_*CUU*_* and acts as an RNA chaperone, possibly with the assistance of additional unidentified cytosolic factors. The interaction causes a conformational change that increases the affinity of *tRNA^*Lys*^_*CUU*_* to another protein factor pre-mitochondrial lysyl-tRNA synthetase (preMsk1p) (Tarassov et al., 1995b), allowing *tRNA^*Lys*^_*CUU*_* to be co-imported into mitochondrial matrix (Brandina et al., 2006; Entelis et al., 2006; Baleva et al., 2017). Moreover, *in vitro* import assays have shown that mitochondria isolated from HepG2 cells can internalize yeast *tRNA^*Lys*^_*CUU*_* (*tRK1*) in the presence of yeast cytosolic factors, suggesting that the mitochondrial RNA import machinery might be conserved in yeast and humans (Kolesnikova et al., 2000). In contrast, yeast mitochondrial import of *tRNA*^*Gln*^ does not seem to require any cytosolic factor (Rinehart et al., 2005), and neither does import of *tRNA*^*Gln*^ into mammalian mitochondria (Rubio et al., 2008).

Cytosolic protein factors have also been shown to regulate the mitochondrial import of *5S* rRNA in mammals. Newly translated mitochondrial ribosomal protein L18 may bind and reshape the conformation of cytosolic *5S* rRNA (Smirnov et al., 2011). This conformational change allows *5S* rRNA to interact with newly synthesized rhodanese, and subsequently the RNA-protein complex is imported into mitochondrial matrix (Smirnov et al., 2010). *In vitro* import of *5S* rRNA into purified mitochondria, however, does not require any of these protein partners (Wang et al., 2010), suggesting that these protein factors are dispensable for mitochondrial import of *5S* rRNA.

In addition to co-importing with cytosolic factors, bringing non-coding RNAs to the close proximity of mitochondrial outer membrane seems to be another way of assisting their import. Most of the nucleus-encoded mitochondrial inner membrane proteins are translated by the ribosomes on the outer surface of mitochondrial outer membrane, and are imported co-translationally (Holt and Bullock, 2009; Williams et al., 2014). A mitochondrial RNA degradation machinery consisting of RNASET2 as the ribonuclease and PNPASE as an RNA transportation regulator has recently been identified in the mitochondrial IMS (Liu et al., 2017). It has been shown that mitochondrion-associated *28S* rRNA can be targeted for degradation by this RNA degradation machinery (Huang et al., 2018). How the *28S* rRNA is selected for degradation and how it is extracted from the ribosomes and imported into mitochondrial IMS remains to be elucidated.

### Translocation Across the Mitochondrial Outer Membrane

Two classes of channels in the mitochondrial outer membrane, the translocase of the outer membrane (TOM) complex and the voltage-dependent anion channel (VDAC) have been postulated as the channels for mitochondrial RNA import.

In yeast *S. cerevisiae*, mitochondrial uptake of *tRNA^*Lys*^_*CUU*_* is inhibited by treatment of mitochondria with proteases, suggesting that certain outer membrane receptors may have a role in *tRNA^*Lys*^_*CUU*_* import (Tarassov et al., 1995a). Furthermore, the mitochondrial cross-membrane translocation of *tRNA^*Lys*^_*CUU*_* is dependent on energy and an intact transmembrane potential, similar to mitochondrial protein import (Entelis et al., 2001). Mitochondria from yeast cells carrying mutant *mom19* or *mim44* alleles show significant defects in *tRNA^*Lys*^_*CUU*_* import, indicating that yeast mitochondrial outer membrane translocase MOM19 and inner membrane translocase MIM44 are important for the import process (Tarassov et al., 1995a).

In plants, cytosolic *tRNA*^*Ala*^ of *A. thaliana* has been shown to interact with *S. tuberosum* VDAC, and its import into isolated mitochondria is inhibited by mitochondrial respiration blockers, such as valinomycin, oligomycin, KCN, and FCCP (Delage et al., 2003). The import can also be inhibited by anti-VDAC antibodies and Ruthenium red, indicating the involvement of VDAC in translocation of tRNAs (Salinas et al., 2006). Moreover, the import is inhibited by trypsin treatment (Delage et al., 2003) and subsequent studies show that TOM20 and TOM40 may have additional roles in regulating tRNA import (Salinas et al., 2006). It should be noted that all these data only show that these proteins are somehow involved, but do not tell whether the involvement is direct or indirect.

### Translocation Across the Mitochondrial Inner Membrane

Some mitochondrial non-coding RNAs may hitchhike the mitochondrial protein import pathway, especially those RNAs that have been shown to be co-imported with mitochondrial proteins such as tRNAs and *5S* rRNA. As mentioned above, in yeast, mitochondrial inner membrane translocase MIM44 has been implicated in *tRNA^*Lys*^_*CUU*_* import (Tarassov et al., 1995a). Even though the inner membrane protein translocons have channels that can accommodate the size of an RNA molecule, they do not seem to be compatible with the predominantly negative charge of the molecule if the RNA is imported in a naked state. However, co-importing with mitochondria-targeted precursor proteins may overcome this incompatibility.

The dependence on membrane potential for RNA import varies between organisms. The membrane potential is required for the import of tRNAs into plant mitochondria and the import of *tRNA^*Lys*^_*CUU*_* into yeast mitochondria (Kolesnikova et al., 2000; Delage et al., 2003). However, tRNA import into protozoa *L. tarentolae* and mammalian mitochondria does not need a membrane potential (Rubio et al., 2000, 2008).

In *Leishmania tropica*, tRNA import across the mitochondrial inner membrane involves a 600-kDa RNA-import complex (RIC), and some of the subunits are members of the respiratory complex (Salinas et al., 2008). Whether this inner membrane protein complex exists in other species remains to be clarified.

In mammals, translocation of lncRNAs across the mitochondrial outer and inner membrane is facilitated by mitochondrial IMS protein PNPASE. Mammalian PNPASE localizes on the outer surface of mitochondrial inner membrane (Chen et al., 2006), forming a trimer or a dimer of trimers (Wang et al., 2010). It is unlike most peripheral membrane proteins, as the association with the inner membrane is much stronger. PNPASE is a 3′–5′ exoribonuclease in bacteria. PNPASE purified from mammalian mitochondria, however, has no ribonuclease activity (Liu et al., 2017). In fact, neither mitochondrial matrix, nor mitochondrial total membrane that harbors mammalian PNPASE contains any obvious ribonuclease activity (Liu et al., 2017). PNPASE has been shown to not only mediate the import of most lncRNAs and one miRNA *miRNA-378* into mitochondrial matrix, but also the export of mitochondrial RNAs from the matrix to the IMS to be degraded (Wang et al., 2010; Liu et al., 2017; Shepherd et al., 2017). In addition, import of cytosolic *28S* rRNA into mitochondrial IMS for degradation (Huang et al., 2018) and export of *TERC-53* are also mediated by PNPASE (Cheng et al., 2018).

Identifying the RNA channel in the mitochondrial inner membrane remains one of the biggest hurdles of the field. Research done on mtDNA import and export may provide some clues. DNA import into plant mitochondria has been shown to be inhibited by the adenine nucleotide translocase (ANT) inhibitor atractyloside, suggesting the involvement of ANT in DNA import (Koulintchenko et al., 2003). ANT proteins are the main components of the mitochondrial permeability transition pore (mPTP). The effectors of mPTP, however, have opposite effects on DNA import (Koulintchenko et al., 2003), suggesting that ANT itself but not mPTP is involved in DNA import into plant mitochondria. It should be noted that these are all indirect proofs.

Mitochondrial permeability transition pores, however, have been shown to be involved in the release of mtDNA into the cytosol under stress conditions (Oka et al., 2012; Jeonghan et al., 2019). Whether ANTs or mPTPs directly function in mitochondrial RNA translocation across the inner membrane remains to be investigated.

The mitochondrial translocation mechanisms of other mammalian non-coding RNAs, such as *SAMMSON*, *GAS5*, and mecciRNAs, remain largely unclear.

## Mitochondrial RNA Export

Studies have shown that mitochondria also export non-codings RNAs and mtDNA (Table 2). Under stress, mitochondria release mtDNA into the cytosol, which can trigger the immune response by activating the cGAS-STING pathway (Oka et al., 2012; Jeonghan et al., 2019). The mPTP and the outer membrane pore formed by VDAC oligomerization have been shown to be involved in the release of mtDNA (Oka et al., 2012; Jeonghan et al., 2019). Recent studies have shown that mitochondria also release double-stranded RNAs (dsRNAs) into the cytosol in a PNPASE-dependent manner (Dhir et al., 2018). It was proposed that PNPASE was the ribonuclease that degrades mitochondrial RNAs in the mitochondrial IMS but not in the matrix, and that defects in mitochondrial RNA degradation led to accumulation of mitochondrial dsRNAs in the cytosol (Chen et al., 2006). PNPASE localizes mainly in the mitochondrial IMS, and so do most mitochondrial nuclease activities such as RNASET2, REXO2 and Endonuclease G (Cote and Ruiz-Carrillo, 1993; Francesco et al., 2013; Huang et al., 2018). It should be noted that mammalian PNPASE has no ribonuclease activity, and neither does mitochondrial total membrane that harbors PNPASE (Liu et al., 2017). Therefore, it is more logical to conclude that PNPASE is involved in the substrate delivering step of mtRNA degradation.

**TABLE 2:** Mammalian mitochondrial RNA exportome.

**Name**	**Mitochondrial function**	**Proposed function outside mitochondria**	**References**
mtDNA	Mitochondrial genome	Initiating immune response	Jeonghan et al., 2019; Oka et al., 2012
*SncmtRNA*	Unknown	Related to cell proliferation and cell cycle?	Birgit et al., 2007; Burzio et al., 2009; Dietrich et al., 2015; Vidaurre et al., 2014
*ASncmtRNA-1/2*	Unknown	Functioning as tumor suppressors?	Birgit et al., 2007; Burzio et al., 2009; Dietrich et al., 2015; Vidaurre et al., 2014
*LIPCAR*	Unknown	Biomarker of cardiac remodeling?	Dietrich et al., 2015; Kai-Chien et al., 2018; Kumarswamy et al., 2014
mecciRNA	Unknown	Functioning as mitochondrial protein folding chaperones, facilitating mitochondrial protein import	Liu et al., 2020
*TERC-53*	Unknown	Initiating mitochondrial stress response?	Cheng et al., 2018; Zheng et al., 2019

In addition to dsRNAs, other mtDNA-encoded non-coding RNAs have been detected outside of mitochondria. One example is *SncmtRNA*, a 2374-nucleotide chimeric transcript composed of mitochondrial *16S* rRNA covalently linked to an 815-nucleotide 5′-leader fragment derived from the complementary strand (Birgit et al., 2007). *SncmtRNA* is polyadenylated and forms an 820 bp double-stranded structure with a 40-nucleotide loop (Dietrich et al., 2015). *SncmtRNA* is preferentially expressed in highly proliferating normal and cancer cells (Birgit et al., 2007). Two antisense lncRNAs of *SncmtRNA* (*ASncmtRNA-1* and *ASncmtRNA-2*) have also been identified in normal proliferating cells (Burzio et al., 2009). All three RNAs are present both in mitochondria and in the nucleus (Eduardo et al., 2011), suggesting a mitochondrial export process. *ASncmtRNAs* are downregulated in tumor cells, and a role as tumor suppressors has been postulated (Vidaurre et al., 2014).

Another mitochondrion-encoded lncRNA *LIPCAR* is also a chimeric transcript, formed between the 5′-end of *COX2* and 3′-end of *CYTB*. It was identified in the plasmas from patients with chronic heart failure (Kumarswamy et al., 2014; Kai-Chien et al., 2018). The function of *LIPCAR* remains unclear.

In addition to lncRNAs, mitochondrial genome also produces a set of miRNAs. These miRNAs, however, appear to remain within mitochondria (Ro et al., 2013). Recently, RNA-sequencing has identified hundreds of circular RNAs (mecciRNAs) that are also encoded by the mitochondrial genome (Liu et al., 2020). Unlike mitochondrial genome-encoded miRNA, mecciRNAs have been shown to shuttle in and out of mitochondria (Liu et al., 2020). While in the cytosol, mecciRNAs function as chaperones in the folding of mitochondrial proteins, facilitating their import (Liu et al., 2020).

Finally, one of the imported RNAs *TERC* has been shown to be processed within mitochondria, and the processing product *TERC-53* is exported to the cytosol, where it functions as a downstream indicator of mitochondrial stress (Cheng et al., 2018). Cytosolic *TERC-53* has been shown to be involved in cellular senescence and organismal aging (Zheng et al., 2019).

## The Functions of Mitochondrial Non-Coding RNAs in Communication Between Mitochondria and Other Cellular Compartments

**FIGURE 1:**
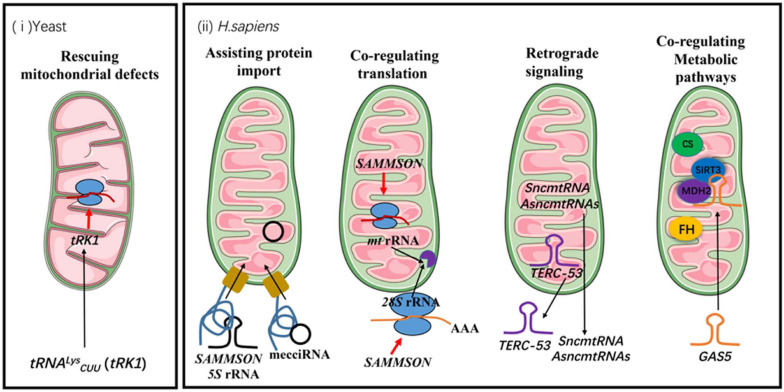
The functions of mitochondrial non-coding RNAs in communication between mitochondria and other cellular compartments. The black arrows indicate the directions of trafficking. The red arrows indicate positive regulations of cellular processes. FH stands for fumarate hydratase and CS stands for citrate synthase.

The known functions of mitochondrial non-coding RNAs in communication between cellular compartments can be roughly categorized into following groups (Figure 1):

### Rescuing Mitochondrial Defects Under Stress

Yeast *tRNA^*Lys*^_*CUU*_* (*tRK1*) is one of the tRNAs that has been shown to be imported into mitochondria (Martin et al., 1979). Yeast mitochondrial genome already encodes a functional *tRNA*^*Lys*^ (*tRK3*). The wobble uridine of the *tRK3* anticodon has a 2-thio modification, which cannot be formed efficiently at 37°C. Consequently, codon recognition by *tRK3* is compromised under heat-shock conditions, and the normally dispensable *tRK1* becomes essential for the translation of *AAG* codon (Martin et al., 1979).

### Assisting Import of Mitochondrial Proteins

For a long time, it was believed that the imported *5S* rRNA was incorporated into mitochondrial ribosomes. However, recent cryo-EM studies show that mammalian mitochondrial ribosomes do not stably integrate *5S* rRNA, but contain a mitochondrion-encoded tRNA instead (Brown et al., 2014; Greber et al., 2015; Rorbach et al., 2016). The function of mitochondrial *5S* rRNA may lie in the import itself. It has been shown that *5S* rRNA is co-imported with rhodanese, acting as a molecular chaperone by interacting with newly-translated rhodanese and maintaining it in an enzymatically inactive state (Smirnov et al., 2010). The interaction facilitates the mitochondrial import of both *5S* rRNA and rhodanese (Smirnov et al., 2010).

Another example of non-coding RNA assisting mitochondrial protein import is *SAMMSON*. *SAMMSON* is predominantly expressed in aggressive melanomas (Leucci et al., 2016). Mitochondrial association of *SAMMSON* has been confirmed with multiplex RNA FISH. Whether *SAMMSON* is imported into mitochondria, however, remains to be confirmed. One of the *SAMMSON*-interacting protein is p32 (Leucci et al., 2016; Vendramin et al., 2018), a well-established regulator of mitochondrial ribosome assembly and mitochondrial protein synthesis (Fogal et al., 2010). Knockdown of *SAMMSON* in melanoma cells impairs mitochondrial targeting of p32, leading to mitochondrial translation defects and subsequently triggering apoptotic cell death (Leucci et al., 2016).

mecciRNAs are the last known group of mitochondrial RNAs that fall into this category (Liu et al., 2020). These circular RNAs can be imported into mitochondria by themselves (Liu et al., 2020). Linearization abolishes their import. mecciRNAs show certain degrees of specificity with the proteins that they assist. The import of the target proteins is most efficient when the RNAs are added co-translationally (Liu et al., 2020). Interaction of the RNAs with known components of mitochondrial protein and RNA import pathways such as TOM40 and PNPASE has also been observed (Liu et al., 2020). One of the mecciRNAs that have been further studied is *mecciDN1*. It has been shown to facilitate the mitochondrial import of RPA32 and RPA70, both of which are components of mtDNA replication and repair machineries (Liu et al., 2020). Under stress conditions such as UV and hydrogen peroxide treatments, *mecciDN1* levels are upregulated and mitochondrial RPA32 and RPA70 levels increase even though the total protein levels remain the same (Yue et al., 2006; Liu et al., 2020). Upregulation of *mecciDN1* levels was also observed in tumor samples.

### Co-regulating Cytosolic and Mitochondrial Translation Programs

Mitochondrial biosynthesis requires coordination of nuclear and mitochondrial genomes. The subunits of OXPHOS complexes are encoded in both genomes, and synchronization of the cytosolic translation and mitochondrial translation programs has been shown to be critical for the assembly of these complexes and their functions (Couvillion et al., 2016). *SAMMSON*-interacting protein p32 in cancer cells is a well-established regulator of mitochondrial ribosome assembly and mitochondrial protein synthesis (Fogal et al., 2010). Knockdown of *SAMMSON* in melanoma cells decreases mitochondrial p32 levels and induces mitochondrial protein synthesis defects (Leucci et al., 2016). Recent studies have shown that *SAMMSON* also regulates rRNA maturation and protein synthesis in the cytosol by interacting with CARF (Vendramin et al., 2018). CARF is normally a nucleoplasm protein that interacts with XRN2 (5′–3′ exoribonuclease 2) (Shigeko et al., 2015). XRN2 has a crucial role in maturation of virtually all RNAs and in nuclear RNA turnover. In the nucleoli, it is vital for maturation of *5.8S* rRNA and *28S* rRNA (Shigeko et al., 2015). Interaction of CARF with XRN2 in the nucleoplasm thus limits entry of XRN2 into the nucleoli. *SAMMSON* promotes p32 binding to CARF in the cytosol, which limits CARF localization in the nucleoplasm, interferes with its binding to XRN2, and favors localization of XRN2 to the nucleoli (Vendramin et al., 2018). Therefore, *SAMMSON* stimulates protein synthesis both in the cytosol and mitochondria, hence conferring a growth advantage to the cancer cells.

A recently discovered RNA degradation machinery in the mitochondrial IMS consisting of RNASET2 as the ribonuclease and PNPASE as an RNA transportation regulator has also been shown to co-regulate cytosolic translation and mitochondrial translation, but in a different fashion (Liu et al., 2017; Huang et al., 2018). Most of the nucleus-encoded mitochondrial inner membrane proteins have been shown to be translated by mitochondrion-associated ribosomes at the outer surface of mitochondrial outer membrane (Holt and Bullock, 2009; Williams et al., 2014). The IMS RNA degradation machinery not only functions in degradation of mitochondrial rRNAs and mRNAs, but also selectively degrades *28S* rRNA from the ribosomes on the outer surface of mitochondrial outer membrane (Liu et al., 2017; Huang et al., 2018). The ribonuclease activity responds sensitively to a series of cellular conditions, such as pH, ATP and divalent metal ions (Liu et al., 2017; Huang et al., 2018), providing an elegant mechanism for co-regulating translation programs in and out of mitochondria, as well as coordinating mitochondrial biosynthesis to these cellular conditions. Moreover, the ribonuclease activity regulates both nuclear rRNA transcription and mitochondrial rRNA transcription through a yet uncharacterized compensatory feedback signaling pathway (Liu et al., 2017; Huang et al., 2018). How the rRNAs are delivered across mitochondrial inner membrane and outer membrane also remains to be investigated.

What we have described are two cases where general translation programs are regulated by mitochondrial non-coding RNAs. There are also mitochondrial non-coding RNAs that regulate expression of individual proteins both inside and outside mitochondria. One example is *miR-1*, a microRNA specially induced during myogenesis (Zhang et al., 2014). *miR-1* is imported into mitochondria, where it recruits *ND1* and *COX1* mRNAs to mitochondrial ribosomes in an AGO2-dependent manner (Zhang et al., 2014). Instead of acting as a suppressor of protein expression, *miR-1* activates expression of ND1 and COX1 in the mitochondria during muscle differentiation (Zhang et al., 2014). *miR-1* has also been shown to target *IGF1* and *CCND1* mRNAs in the cytosol, where it functions as an inhibitor of the expression of these two proteins (Gan et al., 2019). CCND1 is an important cell cycle regulator and IGF1 has functions in many aspects of cell metabolism and proliferation (Montalto and Amicis, 2020). By inhibiting their expression, *miR-1* thus has a negative impact on G1/S phase transition, proliferation and viability of cardiomyocytes. By coordination its functions in and out of mitochondria, *miR-1* thereby fine-tunes the switch of cell types and metabolism programs.

### Functioning as Retrograde Signals

Mitochondria have to change their functional states in response to cellular needs and environmental cues. One of the vital steps is reporting their functional states to the nucleus and other cellular compartments, so mitochondrial biosynthesis, and mitochondrial repair or recycling programs such as mitochondrial unfolded protein responses can be upregulated or inhibited (Li et al., 2021). Mitochondrial non-coding RNAs have also been shown to function in mitochondrial retrograde signaling. One example is the dsRNAs exported from mitochondria. In the cytosol, these RNAs engage a MDA5-driven immune signaling pathway that activates a type I interferon response (Dhir et al., 2018). The mitochondrial IMS degradation machinery that restricts the release of mitochondrial dsRNAs could therefore play a vital role in restricting this auto immune response (Liu et al., 2017; Huang et al., 2018; Maciej et al., 2020). Whether it is also involved in defense against the invasion of RNA viruses remains to be investigated.

Another example of mitochondrial RNAs initiating downstream signals outside of mitochondria is *TERC-53*. *TERC-53* is a cleavage product of telomerase RNA *TERC* by the above-mentioned ribonuclease RNASET2 in the mitochondrial IMS (Cheng et al., 2018; Zheng et al., 2019). After its processing, *TERC-53* is exported and localizes mainly in the cytosol. The cytosolic *TERC-53* engages different downstream effectors in different cell types (Cheng et al., 2018; Zheng et al., 2019). We have shown that in cancer cells and in neuronal cells, cytosolic *TERC-53* has very different binding partners (Unpublished data) (Cheng et al., 2018; Zheng et al., 2019). *TERC-53* levels respond to mitochondrial functional states and one of the downstream events is reprogramming the expression of a subset of genes that are involved in stress responses, which eventually manifests in cellular senescence and organismal aging (Cheng et al., 2018; Zheng et al., 2019).

*SncmtRNA* and its antisense lncRNAs (*ASncmtRNA-1* and *ASncmtRNA-2*) have been found both in mitochondria and in the nucleus (Birgit et al., 2007; Burzio et al., 2009; Vidaurre et al., 2014; Dietrich et al., 2015; Figure 1). Both *ASncmtRNAs* are downregulated in cancer cells, and further knockdown of *ASncmtRNAs* in cancer cells induces cell death by apoptosis. In endothelial cells, *ASncmtRNA-2* is upregulated in aging and replicative senescence (Bianchessi et al., 2015). Even though their exact functions remain unknown, their localization in the nucleus suggests a role in mitochondrial retrograde signaling.

### Co-regulating Metabolic Pathways in Mitochondria and the Cytosol

Most of the functions summarized so far may eventually lead to co-regulation of the cytosolic and the mitochondrial metabolic programs, but some mitochondrial non-coding RNAs appear to directly regulate the activities of the components of these metabolic pathways. *GAS5* is a lncRNA that contains a 5′-terminal oligo pyrimidine (TOP) stretch (Mourtada-Maarabouni et al., 2010). The 5′-TOP sequence is normally found in mRNAs under the control of mTOR (Kahan and Meyuhas, 2015). Recent studies have shown that *GAS5* is translocated to mitochondria under nutrient stress, where it disrupts metabolic enzyme tandem association of fumarate hydratase, malate dehydrogenase, and citrate synthase (Sang et al., 2021). Downregulation of the interaction between *GAS5* and these enzymes has been observed in cancer cells (Sang et al., 2021). Even though the mechanistic details linking *GAS5* and mTOR are still to be elucidated, given the importance of mTOR in nutrient sensing and metabolism, these data suggest a co-regulation of the metabolic pathways in and out of mitochondria by *GAS5*.

## Discussion

There have been some doubts about the validity of the mitochondrial RNA import field, partially due to the technical challenge of truly separating isolated mitochondria and cytosolic contaminants. Other concerns came from the findings that some of the imported RNAs do not share the same functions as their cytosolic counterparts. Opposing evidence has been expertly summarized by Gammage et al. (2018). Most of the arguments have been focused on three non-coding RNAs: *RMRP*, *H1* RNA and *5S* rRNA.

Mitochondrial RNA processing ribonuclease (RNase MRP) was originally identified as a site-specific endoribonuclease that generates primer RNA for mtDNA replication (Chang and Clayton, 1987b). It contains an RNA subunit, *RMRP* (Chang and Clayton, 1987a; Lu et al., 2010). Mitochondrial G-rich RNA sequence-binding factor 1 (GRSF1) has been shown to bind *RMRP* and thus enhance its retention in the mitochondrial matrix (Noh et al., 2016). However, it has also been shown that after micrococcal nuclease treatment of Percoll gradient-purified HeLa mitochondria, the amount of full-length *RMRP* associated with the mitochondria is about 1 RNA molecule per 100 mitochondria (Kiss and Filipowicz, 1992), suggesting that the detected mitochondrial *RMRP* might come from outer membrane-associated contaminants. On the other hand, Topper and colleagues argued that the very low level of *RMRP* should be sufficient for its function (Topper et al., 1992). In addition, *in situ* hybridization and biochemical analysis has also supported mitochondrial localization of *RMRP* in mouse cardiomyocytes (Li et al., 1994). More recently, the necessity of *RMRP* in mitochondria was again brought into question, as it has been reported that the primer RNA required for mtDNA synthesis can be formed by transcription termination stimulated by the G-quadruplex structure of nascent RNA (Wanrooij et al., 2010), independent of RNase MRP. It, however, should be noted that the imported *RMRP* is cleaved and processed (Chang and Clayton, 1987a; Noh et al., 2016), so the quantification of the levels of the RNA within mitochondria might not be accurate and the function is most likely different from its nuclear counterpart.

The controversy on mitochondrial import of *H1* RNA is mostly based on two findings that could easily coexit. Nuclear *H1* RNA is the RNA subunit of RNase P. It forms the catalytic core of the enzyme, and can mediate the cleavage of tRNA precursors at the 5′ end in the absence of protein subunits (Kikovska et al., 2007). The secondary structures of bacterial, archaeal and eukaryotic nuclear *H1* RNAs are conserved (Ellis and Brown, 2009). In yeast mitochondria, *H1* RNA is encoded by mitochondrial gene *RPM1* (Turk et al., 2013), but in mammalian mitochondria, a nucleus-encoded RNA has been shown to be a part of the RNase P activity. The RNA sequence of mitochondrial *H1* RNA appears to be identical with its nuclear counterpart (Puranam and Attardi, 2001), even though the substrate specificities are different (Rossmanith et al., 1995). The controversy arose when a protein-only RNase P complex, comprising of MRPP1, MRPP2, and MRPP3, has been identified in human mitochondria. *In vitro* assays showed that the complex reconstituted with the proteins purified from bacteria is capable of processing single tRNA procursors (Holzmann et al., 2008). Again, it should be noted that some mitochondrial tRNA genes are grouped together and lack an intervening presequence. Removing the RNA component of mitochondrial RNase P activities results in inefficient processing of these tRNA precursors (Wang et al., 2010), suggesting that the two mitochondrial RNase P complexes may coexist and have different substrate specificities.

The debate on *5S* rRNA import into mitochondria mainly stems from a lack of clear mitochondrial function. Even though mammalian mitochondrial genomes encode two ribosomal RNAs (*16S* and *12S* rRNA), nucleus-encoded *5S* rRNA has also been detected in highly purified mammalian mitochondria (Yoshionari et al., 1994; Magalhães et al., 1998). *5S* rRNA is an integral component of ribosomes in nearly all organisms. In plant, *5S* rRNA is encoded by the mitochondrial genome and is a component of plant mitochondrial ribosomes (Waltz et al., 2020). Therefore, it was naturally assumed to be a subunit of the mammalian mitochondrial ribosome. Unexpectedly, *5S* rRNA is absent in the structures of mammalian mitoribosomes. The putative position of *5S* rRNA is occupied by a L-shaped mitochondrial tRNA (Brown et al., 2014; Greber and Ban, 2016). Therefore, the molecular function of mitochondrial *5S* rRNA remains to be identified. Non-coding RNAs having different functions in different locations, however, has been proven to be more of a common theme than an exception (Noh et al., 2016; Zheng et al., 2019).

A few concepts also need to be clarified. Mitochondrion-association means colocalization of these RNAs with mitochondria. These RNAs could be on the outer surface of mitochondrial outer membrane or within the outer membrane. FISH assay is a good approach to verify the association but does not distinguish the two possibilities. Biochemical assays such as ribonuclease treatment and mitoplasting (striping mitochondria of their outer membrane) are usually required to determine whether an RNA is indeed imported into mitochondria. However, nuclease treatment and mitoplasting only enriches those RNAs that are imported into mitochondrial matrix. Biochemically, mitochondrial IMS localization of a certain RNA can only be confirmed if the RNA levels are higher in the mitochondrial IMS than in all the other cellular compartments such as ER, lysosomes and the mitochondrial matrix. Otherwise, it is very hard to prove that it is not from contamination. The same argument goes against the matrix localization of a very small fraction of PNPASE as claimed (Borowski et al., 2013). Combined FISH cryo-EM is the only technique with high enough resolution to directly examine mitochondrial IMS localization of an RNA. However, this technique also works best when the RNA levels are relatively high.

Therefore, the current mitochondrial RNA importome encompasses mostly RNAs imported into mitochondrial matrix. The real mitochondrial RNA importome is likely much bigger. As mentioned above, the trafficking appears to be bi-directional and there are at least three destinations. Some cytosolic RNAs are transported into the matrix, while some mitochondrial RNAs are exported to the cytosol. And some RNAs are imported to mitochondrial IMS. It has been shown that neither the mitochondrial matrix nor the mitochondrial total membrane contains any ribonuclease or nuclease activity under normal conditions (Liu et al., 2017). Mitochondrial IMS is where all the ribonucleases and nucleases reside (Liu et al., 2017; Huang et al., 2018). Therefore, most of the RNAs targeted into mitochondrial IMS are quickly degraded and their transportation has to be examined with indirect approaches.

Mitochondrion-associated *28S* rRNA and some other non-coding RNAs are selectively degraded by the RNASET2 in the mitochondrial IMS (Huang et al., 2018), suggesting the import of these RNAs into the mitochondrial IMS. Degradation of these RNAs responses to conditions such as pH, ATP and divalent metal ions both *in vitro* with purified ribonuclease and *in organello* (Huang et al., 2018), which should be a criterion for deducing whether an enzyme directly functions in a mitochondrial process. Most of the enzymes that have been postulated to directly function in mitochondrial RNA degradation do not meet this criterion and are most likely indirectly involved. Interestingly, this machinery seems to share some similarities with a newly discovered proteolytic pathway that degrades cytosolic protein aggregates (Jin et al., 2017).

In addition, we have performed the import assay of RNAs into isolated mitochondria and mitoplasts. Some RNAs are imported into mitochondria with higher efficiency than into mitoplasts, while some prefer mitoplasts to mitochondria, suggesting that the former group of RNAs may be mostly imported into mitochondrial IMS (unpublished data). Moreover, most of these imported RNAs are cleaved (unpublished data) (Chang and Clayton, 1987a; Noh et al., 2016; Cheng et al., 2018). Therefore, they are unlikely to perform similar functions in mitochondria as their full-length counterparts.

Up until now, few attempts have been made at understanding the RNA export processes, except that PNPASE has been shown to be involved in the export of mitochondrial dsRNAs (Dhir et al., 2018). Most of the studies have been conducted on the import processes. Based on the limited knowledge, import and export pathways may share most of their components. One of the biggest questions is about substrate selection. Because of the complete lack of evidence, we can only speculate that maybe interaction with other proteins might determine which RNAs remain in the matrix.

## Conclusion

Nucleic acid translocation in and out of mitochondria has become an exciting field with lots of challenges. There are still many important questions to be answered. How are these RNAs selected for import and export? Why are some destined for degradation in the mitochondrial IMS but not the other? How do RNAs cross mitochondrial inner membrane? What are the functions of these RNAs? With the recent development of sequencing and imaging technology, more mitochondrial non-coding RNAs are sure to be identified. Caution, however, should be taken when interpreting the results. It is important not to have pre-assumptions. Most of the time, we tend to look for similar functions for a biological molecule in a different location. No similar function found should never be a direct argument against the localization. Given the fact that most of the imported lncRNAs are processed, their functions in mitochondria are most likely different from those of the full-length cytosolic counterparts. On the other hand, a simple correlation study should not be used as the full proof of a direct function.

It has also become increasingly clear that most of these import and export events happen under specific conditions such as nutrient stress and only in certain cell types. Non-coding RNAs normally are involved in fine-tuning of cellular processes. The study on functions sometimes proves to be difficult given the lack of strong phenotypes. Finding the right conditions and right model systems is essential. Finally, it is always important to keep an open mind. It used to be believed by most that mitochondrial RNA degradation could only happen in the matrix, but with more evidence emerging, mitochondrial RNA degradation in the IMS has become more widely accepted even though the identity of the ribonuclease is still under debate.

## Author Contributions

JH and GW contributed in writing and revision of the manuscript. SW and PW contributed in preparing the tables and figure.

## Conflict of Interest

The authors declare that the research was conducted in the absence of any commercial or financial relationships that could be construed as a potential conflict of interest.

## Publisher’s Note

All claims expressed in this article are solely those of the authors and do not necessarily represent those of their affiliated organizations, or those of the publisher, the editors and the reviewers. Any product that may be evaluated in this article, or claim that may be made by its manufacturer, is not guaranteed or endorsed by the publisher.
